# Investigating Driver Fatigue *versus* Alertness Using the Granger Causality Network

**DOI:** 10.3390/s150819181

**Published:** 2015-08-05

**Authors:** Wanzeng Kong, Weicheng Lin, Fabio Babiloni, Sanqing Hu, Gianluca Borghini

**Affiliations:** 1 College of Computer Science, Hangzhou Dianzi University, Hangzhou 310018, China; E-Mails: kongwanzeng@hdu.edu.cn (W.K.); tuoluo2004@163.com (W.L.); sqhu@hdu.edu.cn (S.H.); 2 College of Biomedical Engineering & Instrument Science, Zhejiang University, Hangzhou 310027, China; 3 Department of Molecular Medicine, University of Rome “Sapienza”, Rome 00185, Italy; 4 Department of Physiology and Pharmacology, University of Rome “Sapienza”, Rome 00185, Italy; E-Mail: gianluca.borghini@gmail.com; 5 IRCCS Fondazione Santa Lucia, via Ardeatina, 306, Rome 00142, Italy

**Keywords:** driving fatigue, eeg, granger causality, frequency domain, brain effective network

## Abstract

Driving fatigue has been identified as one of the main factors affecting drivers’ safety. The aim of this study was to analyze drivers’ different mental states, such as alertness and drowsiness, and find out a neurometric indicator able to detect drivers’ fatigue level in terms of brain networks. Twelve young, healthy subjects were recruited to take part in a driver fatigue experiment under different simulated driving conditions. The Electroencephalogram (EEG) signals of the subjects were recorded during the whole experiment and analyzed by using Granger-Causality-based brain effective networks. It was that the topology of the brain networks and the brain’s ability to integrate information changed when subjects shifted from the alert to the drowsy stage. In particular, there was a significant difference in terms of strength of Granger causality (GC) in the frequency domain and the properties of the brain effective network *i.e.*, causal flow, global efficiency and characteristic path length between such conditions. Also, some changes were more significant over the frontal brain lobes for the alpha frequency band. These findings might be used to detect drivers’ fatigue levels, and as reference work for future studies.

## 1. Introduction

Driver fatigue has been identified worldwide as one of the main reasons for traffic accidents. According to the information from NHTSA (National Highway Traffic Safety Administration) of the United States (US), there are about 100,000 crashes caused by driver drowsiness or fatigue annually, and these accidents cause more than 1500 fatalities and 71,000 injuries [1,2,3,4]. In Europe, driver fatigue causes about 6000 deaths every year [5] and many studies claim that the main cause of the 15%–20% of all traffic accidents is driver fatigue [3,6,7]. In 2010, the Ministry of Public Security of the People’s Republic of China reported about 4,000,000 traffic accidents, and the number is still increasing. Mental fatigue accidents do not only exist in ordinary road traffic, but also in airline and railway industries. In these industries, a great part of accidents is due to drivers’ wrong operations caused by fatigue. In 1996, Morris and Miller did an experiment using a flight simulator, and found that pilots would fall into a drowsy status after about four and a half hours, then the possibility they would undertake incorrect operations would significantly increase [8]. Compared with the normal civil field, accidents in these industries would cause much worse outcomes, even a disaster. It would be very helpful to detect drivers’ fatigue levels and provide an alert before it is too late to avoid or at least reduce these accidents.

Driver fatigue is caused by lots of factors, such as stress, long time driving, lack of rest or continuous sleep, and monotonous driving environments [9,10,11], which seriously affect driving performance. It is a very complex mechanism, which is still not fully understood. Driver fatigue is also reflected in many ways, such as increase of a driver’s reaction time, worse vehicle control performance, changes in brain activity, frequency of eye blink/movement, and heart rate. Current related researches could be divided roughly into two classes. The first class is based on a driver’s vehicle control behaviors. For this kind, it is necessary to determine which part of these behavioral changes is caused by driver fatigue and which is caused by traffic conditions and other environmental factors [5]. The second class is based on driver’s physiological signals, such as changes of chemical components in blood and heart rate, and electrical or magnetic signals from brain activity, which could be collected from blood samples, electrocardiogram (ECG), electroencephalogram (EEG) and magnetoencephalography (MEG), respectively [1]. Since methods to collect chemical signals are usually intrusive and equipment to collect magnetic signals is usually huge and expensive, the EEG signal offers high temporal-resolution and the procedure to collect it is quite fast and non-intrusive. As the EEG equipment is cheap and portable, it has been widely used to study many mental and driver fatigue states in real environments, and numerous results reported [4,5,12,13,14,15] showed how the EEG may be one of the most predictive and reliable physiological indicators [16,17].

In the latest 20 years, many researchers have started to study the human brain in terms of interactions among the different brain areas [18,19]. In particular, in the last few years, some researchers have proposed the concept of the *Brain Network*. In a brain network, regions of the human brain (or EEG channels) are treated as *Nodes*, while the link between two nodes are called *Edges*. By the Graph Theory, specific indices are defined to calculate and quantify the connectivity strength between each pair of nodes. Threshold procedures are used with the aim of eliminating spurious connections due to the presence of no-physiological signals (noise). The nodes and the relative edges form network structures, which are called *Brain Networks*. There are two kinds of connectivity that are possible to estimate: *Functional Connectivity* and *Effective Connectivity* [20,21]. These networks are called *brain functional networks* and *brain effective networks,* respectively. Functional connectivity is a statistical measure of the temporal correlations between spatially separated neurophysiologic events (*undirected relationship*). Generally, the indexes used to quantify the functional connectivity are the Pearson correlation, partial correlation, partial coherence, mutual information and synchronization likelihood. The effective connectivity is instead a measure of causal influence from one neural unit to another (*directed relationship*) and it is normally estimated by Granger causality model, dynamic causality model and structural equation model. In the presented study, the brain effective networks, based on spectral Granger causality (GC), have been used to study driver fatigue. Driving accidents caused by fatigue are directly induced by long reaction time, lethargy or other symptoms. It has been reported that consciousness depends on the brain’s ability to integrate information [22,23] and integration is usually better understood with effective connectivity [21]. For example, during non-rapid eye movement sleep, one possible reason for losing consciousness is the breakdown of cortical effective connectivity [24]. Current EEG-based methods to study fatigue detection could not describe the interactions between different brain regions and measure the brain’s ability to integrate information. The Granger-Geweke causality is able to measure the strength of effective connectivity and it has been widely applied to investigate the dynamic relationship between different brain regions [25,26]. The basic idea has been first proposed by Wiener [27], it was successively formalized by Granger using autoregressive model and then Geweke proposed the first spectral decomposition of Granger’s time-domain causality [28,29,30]. Since EEG data contains many different frequency components, Granger-Geweke model has been chosen here to calculate the causality strength of connectivity estimated from the gathered EEG data. In order to figure out the feasibility of using spectral Granger causality to investigate the driver fatigue, and to reduce the complexity of the analysis, only pair-wise spectral Granger causality has been considered in this work.

This study has two main goals: (1) try to find out the differences in terms of EEG pattern between the alert status, at the beginning of a long time driving and the pattern of the drowsy status at the end of the driving experiment by using brain effective network method. These differences could be used as indicators for fatigue detection; (2) try to find out which part of human brain cortex most likely relate to driver fatigue, which could be used to decide the most appropriated EEG electrodes position scheme in successive experiments. The paper is organized as follow. Section 2 introduces the experiment design, the EEG data recording procedure, and the methodology for the estimation of the brain network properties and describes the analysis procedure. Section 3 presents the results and Section 4 the meanings of the obtained results are discussed. Section 5 provides the conclusions for the work developed.

## 2. Material and Methods

### 2.1. Experiment Design

**Figure 1:**
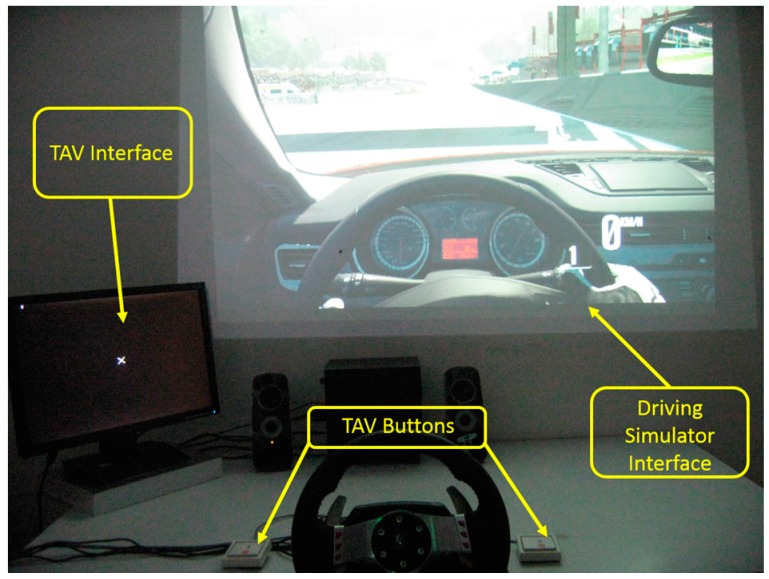
Experimental setup. Note at the bottom of the figure the wheeldrive and beside it the two TAV buttons needed for the execution of the additional tasks other than simple drive (see text for explanations).

A driving simulation experiment has been designed to make the subjects drowsy quickly. In the experiment, a commercial software named *Need for Speed-Shift 2 Unleashed (NFS-S2U)* has been used to simulate the driving environment, since it could provide a quite real visual effect (Figure 1).

In order to achieve the aims of the study, the driving simulation scene was set to the night condition, which was consistent with the real experiment time. In fact, all the experiments were performed between 6 p.m. and 9 p.m., because people are more likely to become tired during this time after daytime work. The selected car was the *Alfa Romeo—Giulietta QV* (1750TBi, 4 cylinders, 235 HP) and the experimental circuit chosen was the Spa–Francorchamps route (Belgium). The experimental protocol included EEG baseline recordings (Eyes Opened—OA; Eyes Closed—OC) and eight driving conditions (*WUP*, *PERF*, *TAV3*, *TAV1*, *TAV5*, *TAV2*, *TAV4* and *DROWSY*). For each condition, subjects were asked to drive two laps of the circuit along the selected track. In the *WUP* (Warm-Up) condition, subjects drove without any requirements, while in the *PERF* (Performance) condition, subjects were asked to reduce the total time by 2% of the previous total time without committing mistakes (e.g., off-road driving). In the *TAVx* conditions, the subjects had to keep track of the total time taken in the PERFO conditions and, at the same time, to execute the *Task of Alert and Vigilance—TAV* (see Section 2.2 for more details). The different levels of the TAV have been used to modulate the difficulty of the global task, and it consists of an alert visual stimulus and a vigilance acoustic stimulus. The difference among the five *TAV’s levels* consisted in the stimulus rate, which is represented by the number *x*, from 1 (EASY) to 5 (HARD). The sequence of *TAVx* conditions was proposed randomly to the subjects to avoid any expectation and habituation effects. In the last experimental condition (*DROWSY),* subjects were asked to drive as in urban centers, that is around a speed limit of 50 km/h. Such monotonous driving conditions were used to induce the drowsy state. After each condition, subjects had to fill out the *NASA-Task Load Index* (NASA-TLX) and the *Karolinska Sleepiness Scale* (KSS) form to collect subjective evaluations of the task workload and driver’s sleepiness [31].

### 2.2. Task of Alert and Vigilance

**Figure 2:**
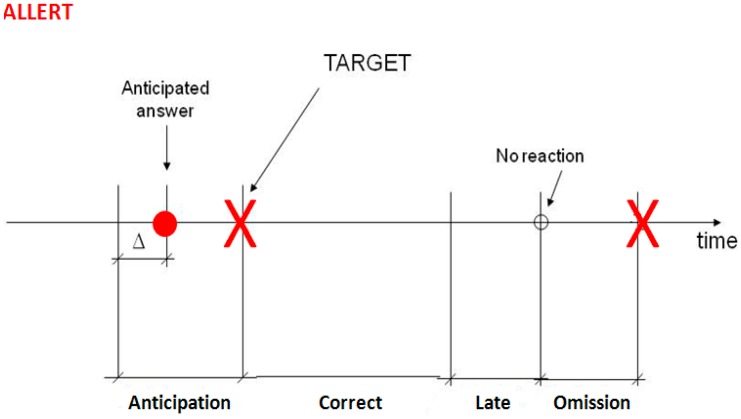
Alert task: The TARGET is the stimulus shown on the screen and the subject has to answer to it as soon as possible. The possible reaction times could be: early, correctly, late or no answer.

The Task of Alert and Vigilance (TAV) was the second task to pay attention at while the subject was performing the driving simulation in the TAV*x* (*x* ranges from 1 to 5) conditions. The *Alert* task (Figure 2) consisted in pressing a button every time an “X” was shown on the screen. It was used to simulate possible obstacles and other cars on real roads. Since the target was presented in the center of the screen, placed 70 (cm) from the user, the subject had 1500 (ms) for pressing the correct button (button number 2) as soon as possible, and the possible reaction times should be “Anticipation”, “Correct”, “Late” and “Omission”.

**Figure 3:**
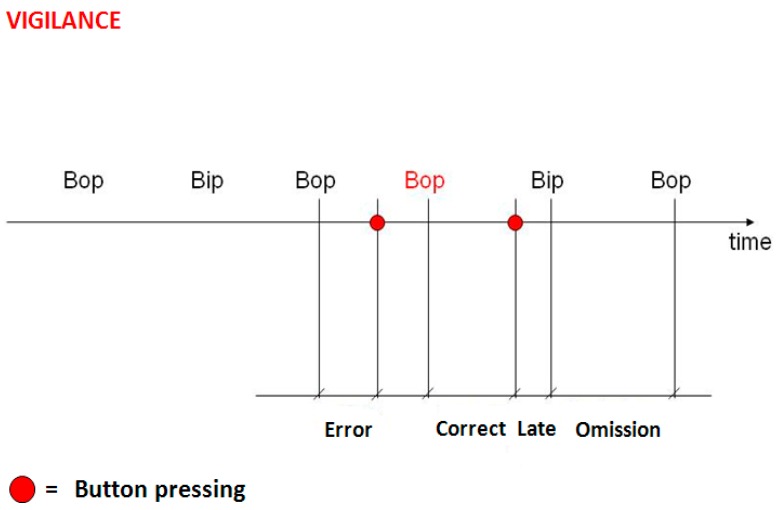
Vigilance task: The target is the identification of the same acoustic tone during the sound sequence, and only after the second identical tone does the subject have to answer by pressing the correct button. If the response is not appropriate, different kinds of errors are provided: early, late, or no answer.

*Anticipation* means that the subject pressed the button before the presentation of the stimulus plus a ∆ time estimated around 350–500 ms. *Correct* corresponds to a reaction in the time interval from the presentation of the “X” to 1500 ms after it, otherwise the answer was classified as *Late* and, if the reaction time exceed 2000 ms, it was classified as *Omission*. In case of “not correct answer”, in the log performance file there was a −1, −2 or −3, respectively, or 0 in case of “correct answer”. In Figure 2, the time definitions of the different answers are shown. At the same time, the subjects had to face the *Vigilance* task (Figure 3) which was the identification of the same acoustical frequency of consecutive acoustic tones (low and high frequency) by pressing the corresponding button (button number 1) before the next tone impulse, with an inter-stimulus time of 2000 (ms). A response after this time interval was named as *Late*, while *Omission* was recorded if there were no answers and *Error* if the response did not coincide with the tone impulses. In Figure 3 is shown an example of a tone sequence and the different types of errors of reaction time. Therefore, one of the tones was used to simulate traffic horns, phone rings, or other occasional sounds which drivers needed to respond to.

### 2.3. EEG Signal Recording

During the entire experiment, scalp EEG signals were recorded by using a 16-channels digital system (*g.tech USBamp*). Fifteen electrodes were placed on the brain according to the 10–20 international system, including *Fz*, *Pz*, *Oz*, *Fp1*, *Fp2*, *F7*, *F3*, *F4*, *F8*, *C3*, *C4*, *P7*, *P3*, *P4*, *P8*. The 16th electrode, placed on the subject’s chest, was used to collect the ECG signal. The ear-lobes were used as reference channels, while the left mastoid as ground channel. The neuro-physiological signals were gathered by a sample rate 256 (Hz). The impedances of the electrodes were kept below 5 kΩ. After each experiment, the recorded signals were checked and EEG data of three subjects were eliminated because of the artifacts. EEG dataset of the WUP and of the DROWSY conditions were selected in line with the aims of the study.

### 2.4. Experimental Subjects

Twelve volunteers students, aged from 23 to 25 (Mean = 23.7; standard deviation = 0.78), right-handed, with regular driving license and no history of neurological diseases, have taken part in the experiment. They had been told to not consume alcohol, tea, coffee or medicine before the experiment. All the subjects were well trained in order to familiarize them with the driving task and the related software interface.

### 2.5. EEG Data Preprocessing and Analysis

In order to improve the signal-to-noise ratio and to remove artifacts (e.g., network frequency interference, muscular artifacts, *etc.*), a preprocessing procedure was applied to the raw EEG data by using the NPX Lab [32] software and some *ad-hoc* Matlab scripts. Raw scalp EEG data were band-filtered within 1–40 Hz and then the *Independent Component Analysis* (ICA) was used to remove the eye-blinks artefacts.

Then, the EEG dataset of each experimental condition was divided into 1 s epochs. The Kwiatkowski-Phillips-Schmidt and Shin (KPSS) test was run for each epoch to check if the data was stationary [33]. Stationary EEG data were used to calculate pair-wise spectral Granger Causality (GC) values in delta band (1–4 Hz), theta band (4–8 Hz), alpha band (8–13 Hz) and beta band (13–30 Hz), for each EEG channel.

The GC values of each epoch were saved into asymmetric matrices. Then, a threshold procedure was applied to the original GC matrixes to eliminate the spurious connections which were likely caused by no-physiological signals that still remained after the preprocessing.

There are, at least, two methods to set the threshold value: One way is by defining a fixed causality strength, the second one is defining a fixed edge percentage, which is also called *Sparsity.* The last method is defined as the ratio between the number of existing edges and the maximum edge number that the brain networks could have. As the GC values are quite different among subjects by the first threshold selection method, it would be quite hard to set a proper threshold value or decide one rule to set different values for all of them. For this reason, the second method has been widely used to explore small-world topology of brain networks [34,35,36] and the edge percentages normally used are 15%–25%, 12.2%–26.7% and 11%–25%. In this study, we have chosen the range of edge percentage (*EdgeP*) from 0.05 to 0.6, with a step of 0.05, which is enough to cover the significant small-world property range [35,37].

Two Matlab toolboxes, Granger Causal Connectivity Analysis (GCCA) and Brain Smart (BSMART), were used to estimate the Granger causality [38,39]. The Brain Connectivity Toolbox (BCT) toolbox was then used to calculate the network properties [40]. See the Appendix for the details on how the Granger Causality was estimated from EEG data.

### 2.6. GC Network Analysis

After the threshold procedure, an *N* × *N* (*N* is number of the nodes in the brain network, *N* = 15 in this paper) directed weighted network ${\text{G}}^{\text{w}}$ could be constructed from one GC matrix. Each node of ${\text{G}}^{\text{w}}$ represents an EEG channel and every directed edge represents an effective connection between two EEG channels. Properties of the GC matrix were calculated according to the formulas described in the following, where ***V*** represents the set of all the nodes of the network ${\text{G}}^{\text{w}}$.

#### 2.6.1. Weighted Degree

In a binary network, the degree of a node describes how many edges are connected to such node, or how many neighbors the node has. For the *i*-th node (node *i*) of a binary directed brain network, its out-degree tells how many edges of the brain network start from node *i* to other nodes, and its in-degree tells how many edges of the network start from other nodes to the node *i* [40]. For weighted brain networks, weighted degree represents the sum of the edges’ strength and the degree of importance of a node; the larger the node’s degree, the more important the node is. Weighted in-degree and out-degree for the *i*-th node of a weighted directed network are written as $k_{i}^{w,in}$, $k_{i}^{w,out}$ respectively, and the formulas to calculate them are as follow:

(1) $$k_{i}^{w,in}=\sum_{j\in V,j\neq i}G^{w}(\vec{j,i})$$

(2) $$k_{i}^{w,out}=\sum_{j\in V,j\neq i}G^{w}(\vec{i,j})$$

In Equations (1) and (2), $\vec{j,i}$ means strength of the direct effective connectivity from node *j* to node *i*, and $\vec{i,j}$ means from node *i* to node *j*.

#### 2.6.2. Characteristic Path Length

Characteristic path length (*L*) is a global property to measure the typical separation between two nodes [41], and is one measure of functional integration in brain networks. *L* is defined as the average of all the shortest absolute path lengths [35,40,42]. In a weighted GC network ${\text{G}}^{\text{w}}$, it means the average strength of the effective connectivity of all the shortest absolute paths. The formula to calculate the L is:

(3) $$L=\frac{1}{N}\sum_{i\in V}\frac{\sum_{j\in V,j\neq i}L_{i,j}}{N-1}$$

where $L_{i,j}$ is the summarized causality strength of the shortest path between node *i* and node *j*.

${\text{G}}^{\text{w}}$ could be binary (by setting all non-zero weighted values to 1) and transformed to a binary network ${\text{G}}^{b}$. Based on the procedure, it is easy to know that ${\text{G}}^{\text{w}}$ and ${\text{G}}^{b}$ are only different at the weight values, besides that they have exactly the same node-set ***V*** and the same edge distribution. Characteristic path length of the corresponding binary network provides the average length of all shortest paths between pairs of nodes.

#### 2.6.3. Global Efficiency

Global efficiency (*Eg*) of a brain network is defined as the harmonic mean of the inverse of the shortest path length of all pairs of nodes in the network [40,43,44]. It measures the communication efficiency of a network, and is one of the properties which could measure the network’s ability to integrate information [40]:

(4) $$E_{g}=\frac{1}{N(N-1)}\sum_{i,j\in V;i\neq j}\frac{1}{L_{i,j}}$$

#### 2.6.4. Causal Flow

Causal flow (*CF*) of a node is defined as the difference between its out-degree and its in-degree [38,45,46]. For weighted network ${\text{G}}^{\text{w}}$, *CF* of node *i* is calculated as follows:

(5) $$CF_{i}^{w}=k_{i}^{w,out}-k_{i}^{w,in}$$

If a node has a highly positive *CF* value, it means granger causal influence from this node to others is much bigger than other nodes to this one. This kind of node is more likely to be the “causal source” of a network. If a node has a quite small negative value, it means this node is largely affected by other nodes. This kind of node is called *Causal Sink* of the brain network [38].

#### 2.6.5. Causal Density

Causal density (*CD*) is a novel measure of causal interactivity, which could reflect the overall causal interactivity and the dynamical “complexity” of a system [46,47,48,49]. *CD* of a network has been defined as the fraction of interactions between different neuronal elements, electrodes or brain regions that are causally significant. For a weighted network ${\text{G}}^{\text{w}}$, *CD* of node *i* is calculated as:

(6) $$CD_{i}^{w}=\frac{1}{N}(k_{i}^{w,in}+k_{i}^{w,out})$$

### 2.7. Statistical Analysis

Repeated measures ANOVA (Confidence Interval, CI = 0.95) and t-tests, with a significant level of α = 0.05, have been used for the statistical validation of the results by using the STATISTICA software (Statsoft). In particular, we have performed two one-way repeated-measures ANOVAs, with the factor CONDITION at eight levels (WUP, PERFO, TAV1 ÷ 5, and DROW), performed separately for the NASA-TLX and the KSS, as independent variables. In addition, we have performed 12 two-tailed paired *t*-tests to investigate the differences between the WUP and DROW conditions in terms of Granger Causality (GC), Global Efficiency (Eg), Path Length (L), Percentage of Unconnected Nodes (PUN), Casual Flow (CF) and Casual Density (CD). In each test, Bonferroni correction has been used for multiple comparisons to avoid the risk of occurrence of Type I errors.

## 3. Results

### 3.1. NASA-TLX and KSS

**Figure 4:**
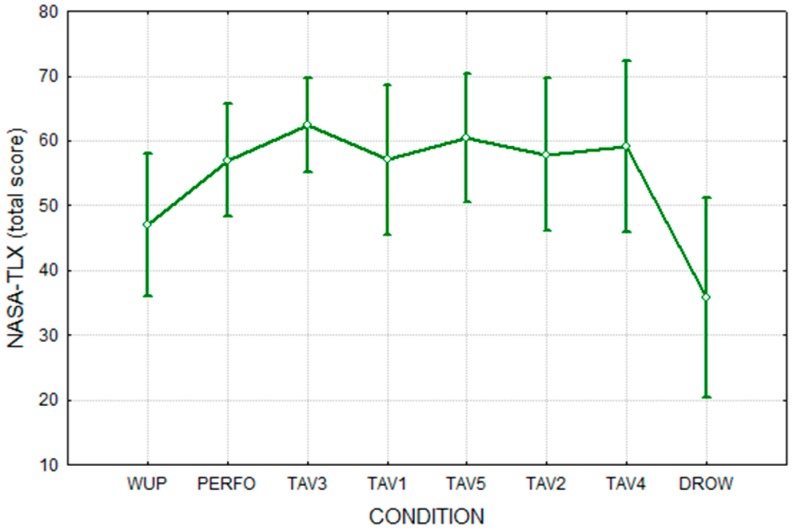
NASA-TLX total score related to the different experimental conditions.

**Figure 5:**
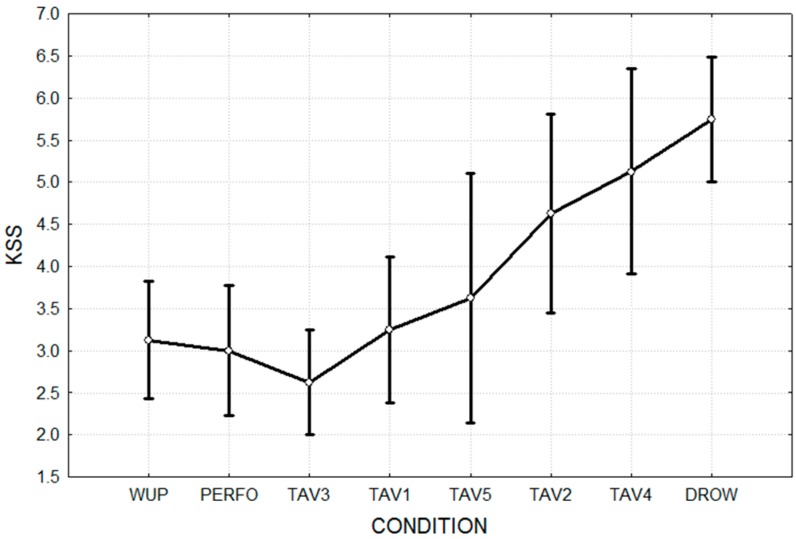
KSS scores related to the different experimental conditions.

The results for the NASA-TLX and of the KSS tests are shown in Figure 4 and Figure 5, respectively. It is possible to note that the DROW condition showed a significantly lower (*p* < 0.05) NASA-TLX score than the WUP condition. In fact, because of the monotonous driving conditions and absence of extra tasks (TAV), the subjects perceived the DROW condition as easier when compared to the other ones. In the same way, since the subjects had a stronger feeling of sleepiness in DROW condition, KSS showed a significantly higher (*p* < 0.05) value with respect to the WUP one.

Since the DROW occurred at the end of the driving protocol and the subjects had to perform many conditions before it, those results were expected. Also, since the WUP and DROW conditions had no additional tasks, they could be considered closer to real driving conditions and with less interference caused by extra tasks. This consideration led us to use the WUP and DROW conditions in the successive EEG analyses.

### 3.2. Granger Causality

**Figure 6:**
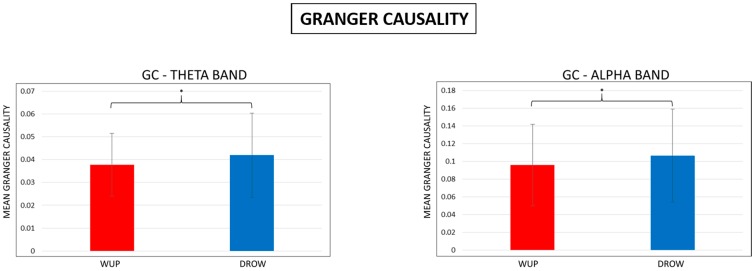
Mean Granger Causality (GC) in the theta and alpha bands. Note that the GC estimated for the DROWSY condition was significantly (*p* < 0.05) higher than the GC in WUP.

In order to check the differences between the WUP and DROW, average values of the GC have been estimated by the spectral GC matrices, and, successively, the four EEG bands in the two conditions were statistically compared. From the t-tests analyses, significant results have been found in the theta and alpha bands (Figure 6). In both the bands, the GC estimated for the DROW (blue bar) was significantly higher than the GC in the WUP (red bar).

### 3.3. Global Efficiency

**Figure 7:**
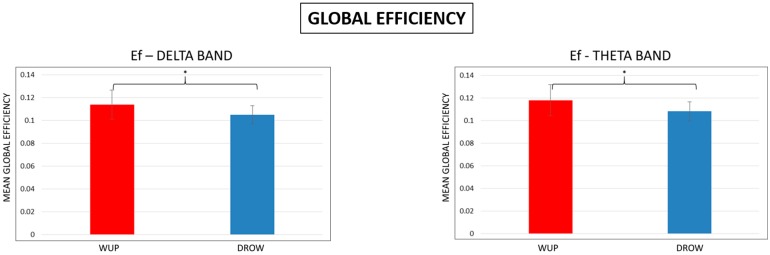
Mean Global Efficiency (Eg) in the delta and theta bands. Note that the Eg estimated for the DROWSY condition was significantly (*p* < 0.05) lower than the Eg in WUP.

After the properties of the brain networks were estimated from the threshold spectral GC matrices, session averages of global efficiency (*Eg*) for the selected conditions were calculated. Results are shown in Figure 7. *Eg* of the DROWSY (blue bar) condition was found to be significantly lower (*p* < 0.05) than the Eg of the WUP (red bar) condition in the delta and theta bands.

### 3.4. Characteristic Path Length and Percentage of Unconnected Nodes

**Figure 8:**
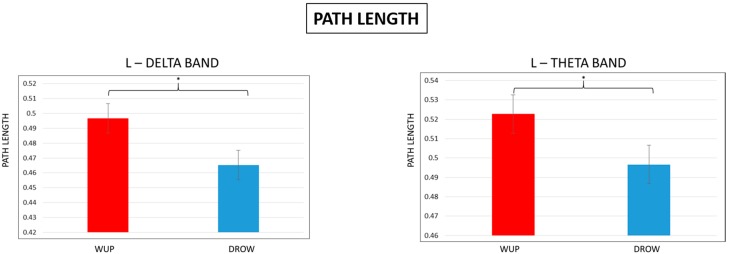
Mean Path Length (L) values in the delta and theta bands. Note that the L estimated for the DROWSY condition was significantly (*p* < 0.05) lower than the L in WUP.

**Figure 9:**
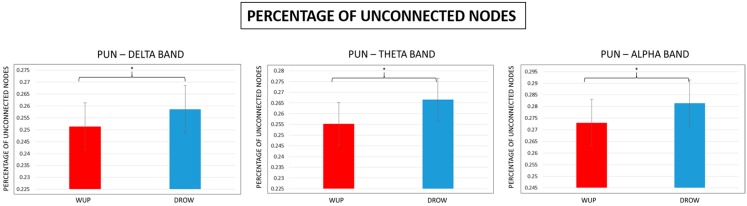
Mean Percentage of Unconnected Nodes (PUN) values in the delta, theta and alpha bands. Note that the PUN estimated for the DROWSY condition was significantly (*p* < 0.05) higher than the PUN in WUP.

According to the definition of *Eg*, characteristic path length (*L*) and the percentage of unconnected nodes (PUN) were further analyzed and compared between WUP and DROWSY conditions in the four EEG bands. Figure 8 and Figure 9 show the results of the bands in which significant differences were found. In particular, Figure 8 reports the results of *t*-tests performed on the *L* data. The plots show that the *L* value estimated in the DROWSY condition was significantly (*p* < 0.05) lower than the *L* value in the WUP both in the delta and theta bands.

In Figure 9, the results of the *t*-tests performed on the PUN values of the two considered conditions are reported. Significant (*p* < 0.05) differences were found in the delta, theta and alpha bands. In particular, in such EEG bands, the PUN estimated in the DROW (blue color) condition was significantly (*p* < 0.05) higher than the PUN calculated in the WUP (red color) condition.

### 3.5. Causal Flow and Causal Density

**Figure 10:**
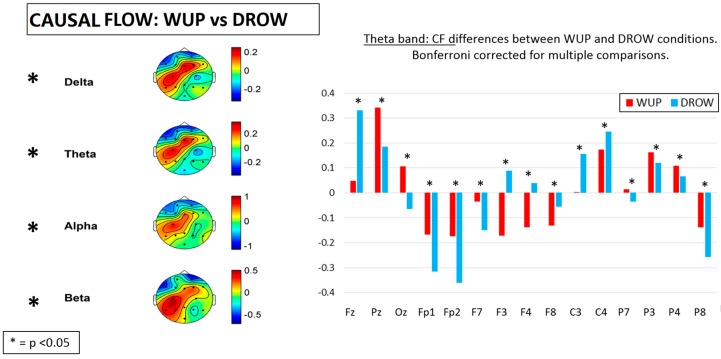
Differences of Causal Flow (CF) between the WUP and DROWSY conditions. Significant differences were found in all considered EEG bands. On the right side of the figure, the mean CF values in the theta band are reported as representative of the others. Note that CF significantly (*p* < 0.05) decreased, especially over the prefrontal, parietal lobes and posterior midline, while significant (*p* < 0.05) increases were found over the frontal and central lobes.

Figure 10 shows the difference between the WUP and DROW conditions in terms of Casual Flow (*CF*) over the different EEG channels. In particular, on the right side of the figure, the mean CF values in the theta band are reported as representative of the other bands. In fact, significant differences (*p* < 0.05) have been found in all the considered EEG bands. The results show that from WUP to DROW condition, a significant reduction of the CF was found over the prefrontal (Fp1 and Fp2) and parietal (P3, P4, P7 and P8) lobes and in the posterior midline (Pz and Oz). On the contrary, significant CF increases were found on the frontal (Fz, F3, F4 and F8) and central (C3 and C4) lobes.

**Figure 11:**
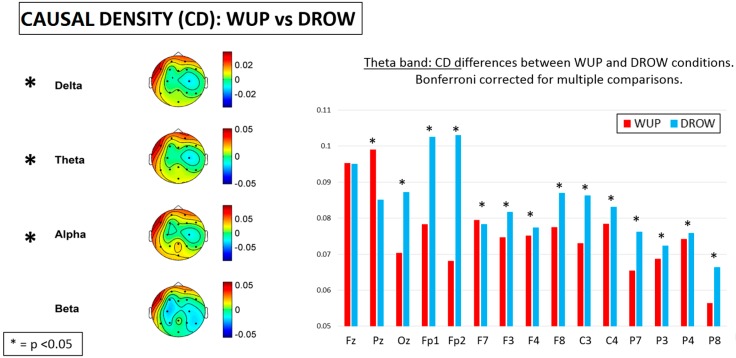
Differences of Causal Density (CD) between the WUP and DROWSY conditions. Significant differences were found in almost all EEG channels for the delta, theta and alpha bands. On the right side of the figure, the mean CD values in the theta band are reported as representative of the others. Note that, from WUP to DROWSY condition, CD significantly (*p* < 0.05) increased over almost all EEG channels.

In Figure 11, the results of the statistical analyses on the Casual Density (CD) data are shown. On the right side of the figure, the mean CD values of the WUP and DROW conditions in the theta band are reported as representative of the others. The difference between the considered experimental conditions provided significant (*p* < 0.05) differences in the delta, theta and alpha bands. In particular, from WUP to DROW, the *CD* increased significantly over almost all EEG channels.

## 4. Discussion

The main results of this study could be summarized as follows: From the WUP to the DROWSY condition, (1) pair-wise Granger causalities (GC) in theta and alpha band increased significantly; (2) in the delta and theta bands, the global efficiency (Eg) and Path Length (L) decreased significantly, and meanwhile the percentage of unconnected nodes increased significantly in delta, theta and alpha bands; (3) *CF* decreased over the prefrontal, parietal and posterior midline and increased over the frontal and central lobes of the brain cortex in delta, theta, alpha and beta bands, while *CD* increased almost over the entire brain in the delta, theta and alpha bands.

Pair-wise granger causalities (GC) are the strength of paths between nodes connected directly, their increase meant that these short connections have been enhanced. Global efficiency (Eg) is one measure of brain’s ability to integrate information. Its decrease indicates that the ability has been weakened from WUP to DROWSY condition. The increase of percentage of unconnected nodes meant that there were significant connection breakdowns from WUP to DROW. This indicates that the topology of the brain network changed, which probably made it harder to transmit information between different brain regions. This could be the reason for the global efficiency reduction observed.

The general increment of the CD index over the brain lobes, in particular over the frontal and central lobes, and the simultaneous reduction of CF index over the prefrontal, parietal and posterior midline, from the WUP to DROW conditions indicated that there was much more information converging to the prefrontal lobes when drivers had fallen into a fatigued state. Therefore, these results might indicate that the information processing speed across such brain regions was affected by driver fatigue.

These phenomena were found to be occurring significantly across different frequency bands, such as delta, theta and alpha, especially over the prefrontal, frontal and parietal lobes. It has been reported that the human brain’s attention and memory performance are related with brain activities in the theta and alpha bands, and the brain’s ability to encode information is correlated with activities in the theta band [50,51]. Reduced ability to concentrate and react on time is common when drivers become drowsy, and it should be reflected by changes in the brain activities in the theta and alpha bands.

## 5. Conclusions

In this paper, differences between alert and drowsy conditions were analyzed in terms of brain networks. Granger-Geweke Causality model in the frequency domain was applied to calculate the effective connections between each pair of EEG channels (nodes). Different brain network properties were determined and compared according to the two considered experimental conditions. Results showed that fatigue causes a reduction in the human brain’s ability to integrate information, which is reflected by a decrease in global efficiency, and that the topology of the whole brain networks also changed, including a variation of the characteristic path length and number of unconnected nodes, which may make it harder to transmit information.

## Appendix

### Granger-Geweke Causality

Granger causality is based on autoregressive models. Assuming that there are two stochastic processes *X* and *Y*, which are assumed to be jointly stationary, the *p*-order autoregressive (AR) model of X and Y could be expressed as:

(A1) $$X_{t}=\sum_{i=1}^{p}a_{1i}X_{t-i}+u_{1t}$$

(A2) $$Y_{t}=\sum_{i=1}^{p}b_{1i}Y_{t-i}+v_{1t}$$

and the *p*-order joint-regressive (JR) model of *X* and *Y* is described as:

(A3) $$X_{t}=\overset{p}{\underset{i=1}{\sum }}a_{2i}X_{t-i}+\overset{p}{\underset{i=1}{\sum }}c_{2i}Y_{t-i}+u_{2t}$$

(A4) $$Y_{t}=\overset{p}{\underset{i=1}{\sum }}b_{2i}X_{t-i}+\overset{p}{\underset{i=1}{\sum }}d_{2i}Y_{t-i}+v_{2t}$$

where *t = 0*, *1*, …, *N*; $a_{1}$, $b_{1}$, $a_{2}$, $b_{2}$, $c_{2}$, $d_{2}$ are the coefficients of the AR model and the JR model, and $u_{1}$, $v_{1}$, $u_{2}$, $v_{2}$ are the zero-mean noises terms. They are uncorrelated with the time and their variances are ${\text{Σ}}_{1}$, $T_{1}$, ${\text{Σ}}_{2}$, $T_{2}$, respectively. According to Granger’s definition, if ${\text{Σ}}_{2}<{\text{Σ}}_{1}$, then *Y* is considered to have a causal influence on X; otherwise if ${\text{Σ}}_{2}={\text{Σ}}_{1}$, *Y* is said to have no causal influence on *X* because it doesn’t make the equation more accurate by adding *Y*. Such causal influence is called *Granger Causality*, and is defined as:

(A5) $${\text{F}}_{\text{Y}\to \text{X}}\text{=ln}\frac{{\text{Σ}}_{\text{1}}}{{\text{Σ}}_{\text{2}}}$$

Similarly, if $T_{2}<T_{1}$, *X* is said to have a Granger causal influence on Y, and the causality is defined as:

(A6) $${\text{F}}_{\text{X}\to \text{Y}}\text{=ln}\frac{{\text{T}}_{\text{1}}}{{\text{T}}_{\text{2}}}$$

If *Fourier* transformation is applied on both sides of the AR and JR model, we can calculate the causality in frequency domain. The causal influence from *Y* to *X* at frequency ω is defined as:

(A7) $${\text{F}}_{\text{Y}\to \text{X}}\text{(ω)=ln}\frac{{\text{S}}_{\text{XX}}\text{(ω)}}{{\tilde{\text{H}}}_{\text{XX}}{\text{(ω)Σ}}_{\text{2}}{\tilde{\text{H}}}_{\text{XX}}^{\text{*}}\text{(ω)}}$$

where $S_{XX}(\text{ω})$ is the auto spectrum of *X*, ${\tilde{H}}_{XX}(\text{ω})$ is the element at position (*X*,*X*) of the normalized form of the transfer matrix $\tilde{H}(\text{ω})$ [29], the star “*” denotes the complex conjugate and matrix transpose. In a similar way, the causal influence from *X* to *Y* should be expressed as:

(A8) $${\text{F}}_{\text{X}\to \text{Y}}\text{(ω)=ln}\frac{{\text{S}}_{\text{YY}}\text{(ω)}}{{\tilde{\text{H}}}_{\text{YY}}{\text{(ω)T}}_{\text{2}}{\tilde{\text{H}}}_{\text{YY}}^{\text{*}}\text{(ω)}}$$

After this step, a series of spectral GC matrixes should contain many spurious connections due to different kinds of noise, which need to be eliminated by the threshold procedure. According to the graph theory, every GC matrix represents a weighted directed brain network.

## References

[1] Yang G., Lin Y., Bhattacharya P. (2005). A driver fatigue recognition model using fusion of multiple features. IEEE Int. Conf. Syst. Man Cybern..

[2] Ji Q., Zhu Z., Lan P. (2004). Real-time nonintrusive monitoring and prediction of driver fatigue. IEEE Trans. Veh. Technol..

[3] Yang G., Lin Y., Bhattacharya P. (2010). A driver fatigue recognition model based on information fusion and dynamic Bayesian network. Inf. Sci..

[4] Lin C.-T., Wu R.-C., Liang S.-F., Chao W.-H., Chen Y.-J., Jung T.-P. (2005). EEG-based drowsiness estimation for safety driving using independent component analysis. IEEE Trans. Circuits Syst. I.

[5] Simon M., Schmidt E.A., Kincses W.E., Fritzsche M., Bruns A., Aufmuth C., Bogdan M., Rosenstiel W., Schrauf M. (2011). EEG alpha spindle measures as indicators of driver fatigue under real traffic conditions. Clin. Neurophysiol..

[6] Dawson D., Fletcher A., Hussey F. (2000). Beyond the Midnight Oil: Parliamentary Enquiry into Managing Fatigue in Transport.

[7] Connor J., Norton R., Ameratunga S., Robinson E., Civil I., Dunn R., Bailey J., Jackson R. (2002). Driver sleepiness and risk of serious injury to car occupants: Population based case control study. BMJ.

[8] Morris T., Miller J.C. (1996). Electrooculographic and performance indices of fatigue during simulated flight. Biol. Psychol..

[9] Brown I.D. (1994). Driver fatigue. Hum. Factors.

[10] Co E., Gregory K., Johnson J., Rosekind M. (1999). Crew Factors in Flight Operations XI: A Survey of Fatigue Factors in Regional Airline Operations.

[11] Rosekind M., Co E., Gregory K., Miller D. (2000). Crew Factors in Flight Operations XIII: A Survey of Fatigue Factors in Corporate/Executive Aviation Operations.

[12] Eoh H.J., Chung M.K., Kim S.-H. (2005). Electroencephalographic study of drowsiness in simulated driving with sleep deprivation. Int. J. Ind. Ergon..

[13] Acharya A., Kar S., Routray A. Phase synchronization based weighted networks for classifying levels of fatigue and sleepiness. Proceedings of the International Conference on Systems in Medicine and Biology.

[14] Chu C.J., Kramer M.A., Pathmanathan J., Bianchi M.T., Westover M.B., Wizon L., Cash S.S. (2012). Emergence of stable functional networks in long-term human electroencephalography. J. Neurosci..

[15] Li W., He Q.-C., Fan X.-M., Fei Z.-M. (2012). Evaluation of driver fatigue on two channels of EEG data. Neurosci. Lett..

[16] Artaud P., Planque S., Lavergne C., Cara H., de Lepine P., Tarriere C., Gueguen B. An on-board system for detecting lapses of alertness in car driving. Proceedings of the 14th International Conference of Enhanced Safety of Vehicles.

[17] Lal S.K., Craig A. (2001). A critical review of the psychophysiology of driver fatigue. Biol. Psychol..

[18] Bullmore E., Sporns O. (2009). Complex brain networks: Graph theoretical analysis of structural and functional systems. Nat. Rev. Neurosci..

[19] Sporns O., Chialvo D.R., Kaiser M., Hilgetag C.C. (2004). Organization, development and function of complex brain networks. Trends Cogn. Sci..

[20] Friston K.J. (1994). Functional and effective connectivity in neuroimaging: A synthesis. Hum. Brain Mapp..

[21] Friston K.J. (2011). Functional and effective connectivity: A review. Brain Connect..

[22] Zeki S., Shipp S. (1988). The functional logic of cortical connections. Nature.

[23] Tononi G. (2004). An information integration theory of consciousness. BMC Neurosci..

[24] Massimini M., Ferrarelli F., Huber R., Esser S.K., Singh H., Tononi G. (2005). Breakdown of cortical effective connectivity during sleep. Science.

[25] Gao Q., Duan X., Chen H. (2011). Evaluation of effective connectivity of motor areas during motor imagery and execution using conditional Granger causality. Neuroimage.

[26] Piantoni G., Cheung B.L.P., van Veen B.D., Romeijn N., Riedner B.A., Tononi G., van Der Werf Y.D., van Someren E.J. (2013). Disrupted directed connectivity along the cingulate cortex determines vigilance after sleep deprivation. NeuroImage.

[27] Wiener N. (1956). The theory of prediction. Mod. Math. Eng..

[28] Granger C.W. (1969). Investigating causal relations by econometric models and cross-spectral methods. Econometrica.

[29] Geweke J. (1982). Measurement of linear dependence and feedback between multiple time series. J. Am. Stat. Assoc..

[30] Dhamala M., Rangarajan G., Ding M. (2008). Analyzing information flow in brain networks with nonparametric Granger causality. NeuroImage.

[31] Kaida K., Takahashi M., Åkerstedt T., Nakata A., Otsuka Y., Haratani T., Fukasawa K. (2006). Validation of the Karolinska sleepiness scale against performance and EEG variables. Clin. Neurophysiol..

[32] Bianchi L., Quitadamo L.R., Abbafati M., Marciani M.G., Saggio G. Introducing NPXLab 2010: A tool for the analysis and optimization of P300 based brain-computer interfaces. Proceedings of the 2nd International Symposium on Applied Sciences in Biomedical and Communication Technologies.

[33] Bressler S.L., Seth A.K. (2011). Wiener-Granger causality: A well-established methodology. Neuroimage.

[34] Bassett D.S., Bullmore E., Verchinski B.A., Mattay V.S., Weinberger D.R., Meyer-Lindenberg A. (2008). Hierarchical organization of human cortical networks in health and schizophrenia. J. Neurosci..

[35] Liu Y., Liang M., Zhou Y., He Y., Hao Y., Song M., Yu C., Liu H., Liu Z., Jiang T. (2008). Disrupted small-world networks in schizophrenia. Brain.

[36] Wu K., Taki Y., Sato K., Kinomura S., Goto R., Okada K., Kawashima R., He Y., Evans A.C., Fukuda H. (2012). Age-related changes in topological organization of structural brain networks in healthy individuals. Hum. Brain Mapp..

[37] Wang J., Wang L., Zang Y., Yang H., Tang H., Gong Q., Chen Z., Zhu C., He Y. (2009). Parcellation-dependent small-world brain functional networks: A resting-state fMRI study. Hum. Brain Mapp..

[38] Seth A.K. (2010). A MATLAB toolbox for Granger causal connectivity analysis. J. Neurosci. Methods.

[39] Cui J., Xu L., Bressler S.L., Ding M., Liang H. (2008). BSMART: A Matlab/C toolbox for analysis of multichannel neural time series. Neural Netw..

[40] Rubinov M., Sporns O. (2010). Complex network measures of brain connectivity: Uses and interpretations. Neuroimage.

[41] Watts D.J., Strogatz S.H. (1998). Collective dynamics of “small-world” networks. Nature.

[42] Watts D.J. (1999). Networks, dynamics, and the small-world phenomenon 1. Am. J. Sociol..

[43] Latora V., Marchiori M. (2001). Efficient behavior of small-world networks. Phys. Rev. Lett..

[44] Latora V., Marchiori M. (2003). Economic small-world behavior in weighted networks. Eur. Phys. J. B.

[45] Liao W., Mantini D., Zhang Z., Pan Z., Ding J., Gong Q., Yang Y., Chen H. (2010). Evaluating the effective connectivity of resting state networks using conditional Granger causality. Biol. Cybern..

[46] Seth A.K. (2005). Causal connectivity of evolved neural networks during behavior. Network.

[47] Seth A.K., Barrett A.B., Barnett L. (2011). Causal density and integrated information as measures of conscious level. Philos. Trans. A Math. Phys. Eng. Sci..

[48] Barrett A.B., Barnett L., Seth A.K. (2010). Multivariate Granger causality and generalized variance. Phys. Rev. E.

[49] Seth A.K., Izhikevich E., Reeke G.N., Edelman G.M. (2006). Theories and measures of consciousness: An extended framework. Proc. Natl. Acad. Sci..

[50] Ray W.J., Cole H.W. (1985). EEG alpha activity reflects attentional demands, and beta activity reflects emotional and cognitive processes. Science.

[51] Klimesch W. (1999). EEG alpha and theta oscillations reflect cognitive and memory performance: A review and analysis. Brain Res. Rev..

